# Does Colonic Diverticulosis Raise the Risk of Colorectal Adenoma in Patients with Colorectal Cancer?

**DOI:** 10.1155/2019/8901026

**Published:** 2019-05-20

**Authors:** Yeon-Ji Kim, Dae Bum Kim, Woo Chul Chung, Ji Min Lee

**Affiliations:** Departments of Internal Medicine, St. Vincent's Hospital, College of Medicine, The Catholic University of Korea, Seoul, Republic of Korea

## Abstract

**Background:**

The aim of this study was to evaluate the risk of development of colorectal adenomas in patients with colorectal cancer (CRC) with and without colonic diverticulosis.

**Methods:**

We performed a retrospective cohort study that included patients with CRC between 2008 and 2011. All patients underwent preoperative colonoscopic and barium enema examinations. Follow-up colonoscopic examinations were performed within 1 year and between 3 and 5 years postoperatively. The incidence of colorectal adenomas was compared based on the presence or absence of diverticulosis. Additionally, multivariate logistic regression analysis was performed to identify the factors independently associated with the development of synchronous and metachronous colorectal adenomas.

**Results:**

Of the 168 patients with CRC included in the study, 55 showed colonic diverticulosis. Synchronous colorectal adenomas were more common in CRC patients with diverticulosis than in those without diverticulosis (*P* > 0.001). Multivariate regression analysis showed that colonic diverticulosis (odds ratio (OR) 3.874, 95% confidence interval (CI) 1.843–8.144, *P* > 0.001) and obesity (body mass index > 25.0 kg/m^2^, OR 2.395, 95% CI 1.089–5.270, *P* = 0.030) were associated with an increased risk of synchronous colorectal adenomas. The presence of synchronous colorectal adenomas increased the risk of metachronous colorectal adenomas (OR 4.407, 95% CI 1.855–10.473, *P* > 0.001).

**Conclusions:**

Colonic diverticulosis was associated with synchronous colorectal adenomas in patients with CRC, which is eventually increasing the risk of metachronous adenomas.

## 1. Introduction

In Western countries, diverticulosis predominantly affects the left-sided colon and is considered an acquired disease. The prevalence of colonic diverticulosis is <10% in the population aged <40 years and increases with age such that approximately two-thirds of the population aged >60 years is affected [1]. Owing to a Western lifestyle and eating habits, the prevalence of diverticulosis has gradually increased in Asians. In contrast to Westerners, Asians show a higher occurrence of right-sided colonic diverticulosis [2, 3]. Colorectal cancer (CRC) is the third most commonly diagnosed cancer worldwide, and >1.2 million patients are newly diagnosed with CRC every year. Disease prevalence increases with advancing age in both colonic diverticulosis and neoplasms. In addition, both diseases share similar disease-causing mechanisms [4, 5]. However, previous studies have not conclusively established an association between colonic diverticulosis and neoplasms.

Barium enema examination is performed to detect colorectal abnormalities including diverticulosis, abnormal colonic movements, colonic narrowing or dilatation, polyps, and cancers. Colonoscopic examination is preferred for the detection and removal of colonic polyps and serves as a standard CRC screening tool. Despite the high diagnostic accuracy, colonoscopic examination could miss left-sided colonic diverticulosis, identified by a barium enema study [6].

We performed both colonoscopic and barium enema examinations to detect colonic diverticulosis in consideration of the false negative causes by one diagnostic method. We evaluated the risk of colorectal adenomas in patients with CRC and investigated the association between colonic diverticulosis and adenomas.

## 2. Materials and Methods

### 2.1. Patients

We performed a retrospective cohort study that included 235 patients who underwent surgical treatment between January 2008 and January 2011 at the Catholic University of Korea, St. Vincent's Hospital. This study was approved by the Institutional Review Board (VC18RESI0175) of the hospital. We enrolled patients who preoperatively underwent both colonoscopic and barium enema examinations for the preoperative detection of synchronous lesions. Patients with CRC were classified into 2 groups—patients with and without diverticulosis. The exclusion criteria were patients aged >85 years and <40 years, patients with advanced-stage cancer (≥stage 4b), patients reporting an incomplete study including failure of endoscopic evaluation for the proximal colon and an incomplete/inconclusive barium enema examination, patients with a history of undergoing screening colonoscopy, patients lost to follow-up, and patients reporting current intake of nonsteroidal anti-inflammatory drugs and low-dose (≤100 mg) and high-dose acetylsalicylic acid.

### 2.2. Preoperative Colonoscopic and Barium Enema Examinations

Colonoscopic and barium enema examinations were performed preoperatively. Colonoscopies were performed by 5 board-certified colonoscopists using a standard video colonoscope (CF240 or CF260, Olympus Optical, Tokyo, Japan). All patients were administered polyethylene glycol-electrolyte powder (COLON LYTE POWD 4 Liter, Meditech Korea Pharm, Seoul, Korea) mixed with 4 liters of water. Colonoscopic examination was performed to evaluate the entire colon for synchronous cancers, adenomas, and diverticulosis. The number, size, and histopathological findings of CRC and colorectal polyps were recorded. Multiple colorectal adenomas were defined as the presence of ≥3 adenomas. Advanced colorectal adenomas were defined as the presence of at least 1 adenoma showing at least 1 of the following features: a colorectal adenoma measuring >1 cm in diameter, tubulovillous or villous adenomas, and/or severely dysplastic lesions. Diverticulosis was defined as the presence of ≥1 diverticula. Based on the locations of diverticula, patients were classified into 3 groups: those with right-sided, left-sided, and bilateral type of diverticular disease. Barium enema examination was performed after the preoperative colonoscopy. The presence, location, and the number of diverticula were recorded by the radiologist. These 2 tests were performed blindly for the examiners, and colonic diverticulosis was detected in one or more positive results. The incidence rates of colonoscopically detected colorectal adenomas were evaluated at specific time points: preoperatively within 1 year and between 3 and 5 years postoperatively.

### 2.3. Postoperative Colonoscopic Examination

Colonoscopic examinations were performed within 1 year and between 3 and 5 years postoperatively. Adenomas detected by colonoscopic examination within 1 year postoperatively were categorized as synchronous adenomas. Follow-up colonoscopic examinations were performed between 3 and 5 years postoperatively for the evaluation of metachronous colorectal adenomas.

### 2.4. Statistical Analysis

Continuous data are expressed as the mean ± standard deviation and were analyzed using an independent sample *t*-test. Categorical variables are expressed as quantities and were analyzed using the *χ* ^2^ test or the Fisher exact test. Logistic regression analysis was used to identify the factors independently associated with the development of colorectal adenomas. All statistical analyses were two-sided, and *P* < 0.05 was considered statistically significant. We used the SPSS statistical package (version 19.0.1, SPSS Inc., Chicago, IL, USA) for all analyses.

## 3. Results

### 3.1. Baseline Patient Characteristics

This study included 168 patients. The mean age of patients was 63.7 years (range 43–3e years), and the mean follow-up was 76.5 months (range 12–ean months). CRC was predominantly located in the left-sided colon: rectum (99, 58.9% patients) and sigmoid colon (40, 23.8% patients). Of 168 patients, 55 (32.7 %) were classified as the diverticular group and 113 (67.2 %) as the nondiverticular group (Table 1). No statistically significant intergroup differences were observed in demographic features including sex, age, location of CRC, body mass index (BMI), and initial cancer stage. There were 35 (63.6%) patients with two or more multiple diverticula, and there was no difference in the presence of a colonic polyp as compared with single diverticulum patients (68.6% vs. 70.0%, *P* = 1.000). Among 55 patients with diverticulosis, 41 (74.5%) patients had right side colon diverticulosis (Table 2). Eighty-six patients were diagnosed with colon polyps by the baseline study. Among them, 62 (72.1 %) patients have colonic polyps located in the right-sided colon including the cecum, ascending colon, and transverse colon.

### 3.2. Diagnosis of Colonic Diverticulosis

Of the 55 patients diagnosed with diverticulosis, 11 patients were diagnosed by both barium enema and colonoscopy. The diagnostic accuracy of colonoscopic examination vs. barium enema examination in detecting colonic diverticulosis was 29.1% (16/55) vs. 90.9% (50/55). Based on the location of the diverticula, patients were classified into 3 groups: those with right-sided, those with left-sided, and those with bilateral type diverticular disease. Right-sided colonic diverticula were observed in 41 (74.5 %), left-sided colonic diverticula in 7 (12.7%), and bilateral colonic diverticula in 7 (12.7%) patients (Table 2).

### 3.3. Synchronous Colorectal Adenomas

We analyzed 89 synchronous colorectal adenomas which included preoperatively diagnosed colorectal adenomas and those diagnosed by colonoscopic examination within a year postoperatively. Logistic regression analysis was performed to investigate the risk for synchronous colorectal adenomas (Table 3). Synchronous colorectal adenomas were more common in CRC patients with diverticulosis than in patients without diverticulosis (*P* > 0.001). Multivariate regression analysis showed that colonic diverticula (odds ratio (OR) 3.874, 95% confidence interval (CI) 1.843–1.843, *P* > 0.001) and obesity (BMI > 25.0 kg/m^2^, OR 2.395, 95% CI 1.089nterval *P* = 0.030) were associated with an increased risk of synchronous colorectal adenomas.

### 3.4. Metachronous Colorectal Adenomas

Follow-up colonoscopic examination was performed in 106 patients 3–5 years postoperatively, and 44 of 106 patients showed metachronous colorectal adenomas. The adenoma detection rates at 3-year and 5-year follow-ups were 17.9 % (19/106) and 23.5% (25/106). Univariate analysis and multivariate regression analysis showed that synchronous colorectal adenomas increased the risk of metachronous colorectal adenomas (OR 4.407, 95% CI 1.855–10.473, *P* > 0.001) (Table 4).

## 4. Discussion

This study showed that colonic diverticulosis and obesity are significantly associated with the risk of synchronous colorectal adenomas. Additionally, synchronous colorectal adenomas showed a significant association with the risk of metachronous colorectal adenomas. Previous studies have reported that the incidence of synchronous and metachronous CRCs was 1.8–19.0% and 0.5–3.6%, respectively [7–10] Most CRCs develop through the stage of colorectal adenomas, which are amenable to early diagnosis and prompt treatment [11–13]. Therefore, early and accurate detection of synchronous colorectal adenomas is important with careful screening of patients at high risk of development of metachronous colorectal adenomas.

Our results showed that colonic diverticulosis and obesity increased the risk of development of synchronous colorectal adenomas. The association between colonic neoplasms and obesity was similar to previous studies [14, 15]. However, the previous several studies that reported an association between colonic neoplasms and diverticulosis remain controversial [16–19]. A study showed that patients with colonic diverticula are not at an increased risk of colorectal adenomas [20], whereas a recent single-center study reported that patients with diverticular disease are more likely to demonstrate colorectal polyps [21]. Despite being addressed by several studies, the association between colonic diverticulosis and neoplasms remains inconclusive. There are potential limitations in previous studies describing this association. First, the diagnostic value of colonoscopic examination for colonic diverticular disease was relatively low, and the diagnostic accuracy of data is questionable. Second, colonic diverticulitis occurs in approximately 20% of cases presenting with colonic diverticulosis. In most studies, colonic diverticulosis was identified based on medical records at the time of hospital admission, and it is unlikely that subclinical events were ruled out completely [22, 23]. Owing to this uncertainty regarding the diagnosis, the association between diverticular disease and colonic carcinogenesis might be underestimated. We performed both colonoscopic and barium enema examinations for better diagnostic accuracy. Our study demonstrated that the diagnostic accuracy of barium enema examination for the detection of colonic diverticula is higher than that of a colonoscopy. Diverticulosis was diagnosed in 32.7% of patients with CRC, and this result was higher than that reported by previous studies about the prevalence of the Korean population.

Colonic neoplasia and diverticulosis share common epidemiological and pathological characteristics. A potential hypothesis that explains the association between colonic neoplasia and diverticulosis is that both diseases are linked to chronic inflammation of the colonic mucosa [24, 25]. Previous studies have suggested an upward shift in cellular proliferation in the colonic mucosa of patients with symptomatic and asymptomatic diverticular diseases [26], which could be related to changes in the intraluminal microenviroment with a subsequent alteration in the immune response. Furthermore, recent studies evaluating the pathophysiology of diverticular disease have observed changes in chemicals or neurotransmitters affecting bowel movements. This observation is supported by the hypothesis that neurotransmitter abnormalities can cause disturbances in bowel movements resulting in a diverticulum-like condition. Serotonin, nitric oxide, and acetylcholine are known neurotransmitters that may contribute to the development of diverticula and may be linked to the development of colonic carcinogenesis [27–30].

Multivariate analysis did not show any association between metachronous colorectal adenomas and colonic diverticulosis. Nevertheless, because metachronous adenomas are eventually associated with synchronous polyp, it is likely that metachronous adenoma will increase if there is colonic diverticulosis. A recent meta-analysis assessing the risk factors for metachronous CRC or polyps showed that the presence of synchronous colonic polyps served as a risk factor for metachronous lesions, which concurs with our results [31]. Therefore, colonic diverticulosis may be associated with the development of metachronous colorectal adenomas in patients with CRC given the observation that colonic diverticulosis increases the risk of synchronous colorectal adenomas.

Colonoscopic examination scores over other screening tests in the accurate detection of colonic polyps and CRC. However, it shows limitations in the evaluation of colonic diverticula [6]. Endoscopists tend to overlook colonic diverticulosis in patients who show lesions suspicious for cancer. The diagnostic value of colonoscopic examination for diverticula detection is affected by colonic anatomy including haustrations, angulations, and preoperative colonic preparation. Right-sided colonic diverticula and polyps are frequently located at the posterior to the haustral folds and on the inner curvature of colonic flexures, which can limit detection of lesions using a forward-viewing colonoscope. Relatively, the diagnostic accuracy of barium enema examination for the detection of colonic diverticula appears to be higher than that of colonoscopic examination [32, 33].

This study is the first to use 2 screening modalities to establish an accurate diagnosis of colonic neoplasia and diverticulosis. We observed that approximately one-third of the patients diagnosed with CRC showed colonic diverticula. Among these, only 29% of patients were diagnosed based on colonoscopic examination indicating the high probability of missing a significant number of diverticula if only colonoscopic examination was to be performed for the diagnosis of diverticula.

This study has several potential limitations. First, we cannot ignore the possibility that a selection bias may have influenced the results owing to the retrospective design. Second, approximately one-third of the included patients were lost to follow-up and did not undergo colonoscopic examination 3–5 years postoperatively. Finally, these results cannot be applied to patients in Western countries because this is a single-institution study performed in Asia. Nevertheless, this is a useful study that accurately diagnosed diverticulosis and analyzed its relationship with adenomas.

## 5. Conclusions

In conclusion, CRC patients with colonic diverticulosis showed a significantly high risk of synchronous colorectal adenomas. Additionally, synchronous colorectal adenomas increased the risk of metachronous colorectal adenomas. Therefore, meticulous colonic examination is warranted in patients with CRC and concomitant diverticulosis.

## Figures and Tables

**Table 1:** Characteristics of patients with colorectal cancer according to the presence of diverticular disease.

	Colorectal cancer without diverticulosis (*N* = 113)	Colorectal cancer with diverticulosis (*N* = 55)	*P* value
Sex (male)	64 (56.6%)	39 (70.9%)	0.107
Age	62.6 ± 11.0	63.9 ± 10.6	0.464
Mean follow-up period	64.1 ± 33.6	66.2 ± 31.2	0.716
Location of colon cancer			0.275
Cecum/ascending colon	12 (10.6%)	9 (16.4%)	
Transverse colon	5 (4.4%)	0 (0.0%)	
Descending colon	1 (0.9%)	2 (3.6%)	
Sigmoid colon	28 (24.8%)	12 (21.8%)	
Rectum	67 (59.3%)	32 (58.2%)	
Body mass index (kg/m^2^)	24.4 ± 3.8	24.8 ± 3.0	0.441
Smoking	24 (21.2%)	14 (25.5%)	0.677
Alcohol	28 (24.8%)	15 (27.3%)	0.873
Colorectal cancer (TNM stage)			
T			0.625
T1	16 (4.2%)	9 (16.4%)	
T2	34 (30.1%)	18 (32.7%)	
T3	56 (49.6%)	22 (40.0%)	
T4	7 (6.2%)	6 (10.9%)	
N			0.242
N0	67 (59.3%)	38 (69.1%)	
N1	34 (30.1%)	15 (27.3%)	
N2	12 (10.6%)	2 (3.6%)	
M			0.744
M0	106 (93.8%)	53 (96.4%)	
M1	7 (6.2%)	2 (3.6%)	
Synchronous colorectal adenoma	49 (43.4%)	40 (72.7%)	>0.001
Multiple colorectal adenoma	18 (15.9%)	13 (23.6%)	0.227
Advanced colorectal adenoma	25 (22.1%)	15 (27.3%)	0.462

**Table 2:** Detection methods and characteristics of diverticular disease.

	Diverticular group (*N* = 55)
Detection methods	
Colonoscopy	16 (29.1%)
Barium enema	50 (90.9%)
Location of diverticulum	
Right	41 (74.5%)
Left	7 (12.7%)
Both	7 (12.7%)
Number of diverticulum	
Single	20 (36.4%)
Multiple (>2)	35 (63.6%)

**Table 3:** Univariate and multivariate analyses for risk of synchronous colorectal adenoma (*n* = 168).

	Univariate analysis			Multivariate analysis		
	OR	95% CI	*P* value	OR	95% CI	*P* value
Sex (female)	0.577	0.309-1.080	0.085			
Age						
≥60	0.714	0.378-1.350	0.300			
<60 (reference)	1.000					
Body mass index (kg/m^2^)						
<23.0 (reference)	1.000					
23.0–25.0	1.458	0.653-3.253	0.358	1.289	0.529-3.141	0.576
>25.0	2.355	1.143-4.850	0.020	2.395	1.089-5.270	0.030
Smoking	1.983	0.934-4.212	0.072	2.150	0.955-4.844	0.065
Alcohol	1.958	0.955-4.018	0.064			
T stage						
1 (reference)	1.000					
2	0.786	0.301-2.049	0.622			
3	0.827	0.334-2.046	0.681			
4	1.768	0.428-7.299	0.431			
N stage						
0 (reference)	1.000					
1	1.595	0.803-3.169	0.183	2.035	0.953-4.345	0.066
2	2.750	0.811-9.325	0.104	3.456	0.943-12.674	0.061
M stage						
0 (reference)	1.000					
1	3.287	0.662-16.310	0.145			
Multiple colorectal adenomas	1.287	0.585-2.831	0.530			
Advanced colorectal adenoma	1.667	0.805-3.451	0.167			
Diverticulum	3.483	1.729-7.017	>0.001	3.874	1.843-8.144	>0.001

**Table 4:** Univariate and multivariate analyses for risk of metachronous colorectal adenoma (*n* = 106).

	Univariate analysis			Multivariate analysis		
	OR	95% CI	*P* value	OR	95% CI	*P* value
Sex (female)	0.447	0.198-1.013	0.052			
Age						
≥60	0.627	0.282-1.394	0.250			
<60 (reference)	1.000					
Body mass index (kg/m^2^)						
<23.0 (reference)	1.000					
23.0-25.0	0.516	0.173-1.535	0.234			
>25.0	1.400	0.576-3.402	0.458			
Smoking	1.256	0.503-3.141	0.625			
Alcohol	1.487	0.639-3.458	0.356			
T stage						
1 (reference)	1.000					
2	0.711	0.226-2.241	0.561			
3	0.329	0.108-1.008	0.052			
4	1.600	0.231-11.082	0.634			
N stage						
0 (reference)	1.000					
1	0.611	0.243-1.536	0.295			
2	1.720	0.358-8.259	0.498			
M stage						
0 (reference)	1.000					
1	4.463	0.449-44.409	0.202			
Multiple colorectal adenomas	1.732	0.638-4.700	0.277			
Advanced colorectal adenoma	1.044	0.426-2.560	0.924			
Synchronous colorectal adenoma	4.407	1.855-10.473	>0.001	4.407	1.855-10.473	>0.001
Diverticulum	2.012	0.889-4.553	0.091			

## Data Availability

The data used to support the findings of this study are included within the article.

## Supplementary Materials

Supplementary Materials This file contains data of the patients' characteristics, presence of colonic diverticulosis colonoscopically detected and barium enema-detected diverticulosis, and presence of synchronous and metachronous polyps.
